# Reactivation fails to offer the improvement sleep does

**DOI:** 10.1093/sleepadvances/zpaf035

**Published:** 2025-05-24

**Authors:** Murray M Barsky, Alexandra Morgan, Robert Stickgold

**Affiliations:** Harvard University, Cambridge, MA 02138USA; Beth Israel Deaconess Medical Center, Boston, MA 02215USA; Beth Israel Deaconess Medical Center, Boston, MA 02215USA; Harvard Medical School, Boston, MA 02215USA

**Keywords:** Sleep, Memory, Memory consolidation, Learning, Probabilistic learning, Prospective randomization, Reactivation, REM sleep, Retroactive interference, Wake reactivation

## Abstract

In a dynamic process that ultimately affords memories their persistence, memory reconsolidation can serve to strengthen associations following reactivation, particularly in sleep, where active processes may effect overnight enhancement. Reactivation can also occur in wake, where improvement would be unexpected. In an earlier study using performance on the Weather Prediction Task (WPT) as a measure of probabilistic category learning, we looked at the effect of sleep and found significant improvement after a daytime nap, where improvement correlated with the amount of REM sleep obtained. When we introduced interference training following sleep, this REM sleep benefit vanished: post-learning task memory was otherwise preserved. Here, we follow up on these results and test whether reactivation itself—wake reactivation—might be sufficient to induce the improvement found after REM sleep. Our results show that it is not: we saw no improvement on the WPT following reactivation in wake, suggesting sleep may be unique in supporting memory improvement. When we looked at interference effects, we saw unexplained differences between wake and sleep that suggest that while interference is uniformly destabilizing of WPT memories during wake, interference after REM show effects on the memory trace formed during initial learning that are distinctly different from its effects on the subsequently sleep-enhanced memory.

**Significance statement**

The route a memory takes following learning is thought to be punctuated by reactivation—reconsolidation events that can occur during wake or sleep. Yet, only processes active during sleep have been shown to enhance memory. Here, we explored the question, is reactivation alone sufficient to induce the improvement found after sleep? Using a wake reactivation paradigm, we found that it is not: wake reactivation did not enhance memory for a probabilistic learning task; at the same time, we observed a destabilizing response to interference following reactivation in wake. Together, these results suggest that processes distinct from those involved in wake reactivation may be responsible for sleep-dependent memory enhancement.

## 1. INTRODUCTION

divided as the dew,

floating mists, to be rained down and

regathered into a river that flows

and encircles.

[from the preface to *Paterson,* William Carlos Williams]

With an unconscious synthesis that is not yet fully understood, memory processes largely active during sleep can help form connections between seemingly unrelated elements encountered during the day, facilitating insight [1–3]. In the Weather Prediction Task (WPT), a classic probabilistic category learning paradigm that tests implicitly learned probabilistic rules [4], sleep-dependent enhancement has been correlated with the amount of REM sleep obtained closely following learning, adding to the broader evidence that REM facilitates memory abstraction for non-declarative (implicit) memory, and suggestive of the theory that overnight improvement may ultimately be through the formation of schemata representing the rules underlying categorical sets [5–7].

Critical to sleep-dependent enhancement is the reorganization theorized to take place in the period after learning and before final consolidation during which a memory is inherently still labile [8]. Classically, lability can be probed with a test of interference: memories that are labile are susceptible to the deleterious effects of interference, while memories that are consolidated show no interference effects [9].

A broader examination of memory consolidation suggests that memory may be better described as being in an ongoing state of reorganization, with relatively brief periods of *reactivation* and reconsolidation followed by relatively longer periods of consolidation—in what might be called *memory evolution* [7, 8]. While initial encoding can be accomplished in milliseconds and initial stabilization over 4 hours, memory can persist for years in a state of relative consolidation. Reactivation that leads to destabilization can occur at any subsequent time, whether by automatic processes or deliberate recall, and within a timeframe of from seconds to minutes can destabilize memories for up to 6 hours for a motor task [10] or 10 hours on a semantic memory task [11]. Memory subject to cycles of consolidation and reconsolidation across time and in both sleep and wake can be variously affected, yet sleep appears to be unique in supporting memory enhancement [7, 12, 13]. The reconsolidation hypothesis suggests that during wake, sleep, and experimentally induced targeted memory reactivation [14], memory consolidation proceeds through a sequence of reactivation, transient destabilization, and subsequent reconsolidation, in repeated sequences that influence the ongoing process of memory e following initial learning [15, 16]. The direct mechanisms for these changes is unknown, though sleep has been seen to accelerate reconsolidation [11].

**Figure 1.**
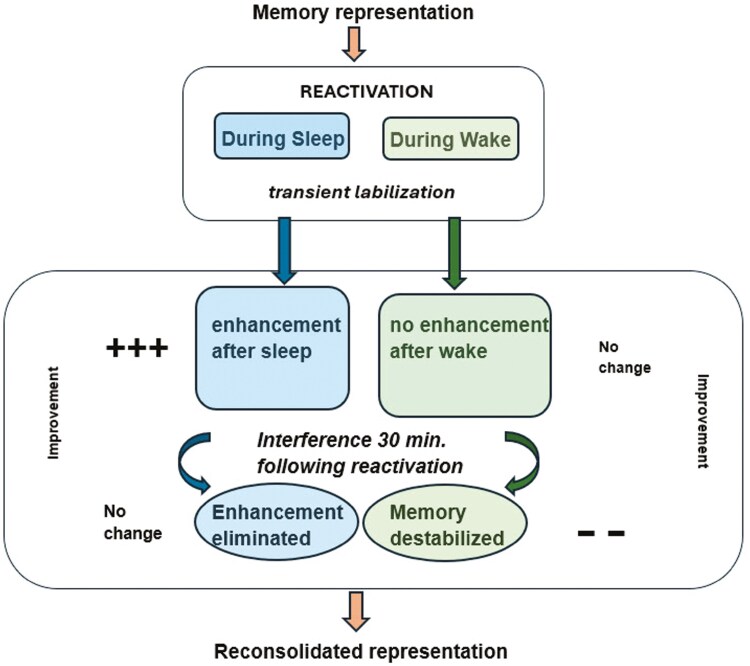
Sleep vs. Wake in memory reactivation; Weather Prediction Task (WPT). Theoretical model. Changes to memory after reactivation can be constructive or destructive, yet only sleep shows evidence of supporting memory enhancement. Interference applied after reactivation can have differential effects after sleep or wake. Improvement estimates (+, -, or no change in adjusted score from initial test to testing following training) based on results from study data utilizing the WPT [19].

Expanding on early work by Walker on sleep-dependent improvement in procedural memory [13], broadening research provides evidence in support of the reconsolidation hypothesis across both sleep and wake and among various memory classifications [17, 18], where upon reactivation, it is thought that previously consolidated memories undergo periods of instability (labilization), allowing the potential for further changes before reconsolidation. During each recurring period of labilization, memories are thought to become permissive to either the positive formation of new associations or to the negative effects of interference [8, 11, 17, 18]. A sleep/wake model, informed by the results of separate experiments with probabilistic memory utilizing the WPT, is shown in Figure 1.

The WPT is a largely non-declarative task in which improvement has been associated with REM sleep [20–23]. For the present study, we focus on the reactivation and interference effects on the “wake” side of Figure 1, with the principal purpose of providing evidence to support at least a partial answer to the question of whether reactivation during REM is unique in supporting memory enhancement.

In the WPT, a set of four cards with line drawings of simple objects serve as stimuli, and participants see one, two, or three cards at each trial, which they use to predict weather outcomes of “sun” or “rain.” Each card has a fixed probability of predicting each outcome, and the probabilities associated with multiple-card combinations are derived from the combined probabilities of the individual cards, but participants are not told of this rule. Rather, after a large number of presentations, participants arrive at an apperception of the relationship between individual cue cards and their likely outcome [15]. Task performance on the WPT represents the percent of responses scored “correct” as determined by comparison to the “sun” or “rain” weather choice that has the highest probability of occurring for each displayed card combination.

Functional imaging studies show that changes occur in distinct brain regions in concert with learning the WPT: the medial temporal lobes are normally active during initial trials but become less active as the basal ganglia become increasingly active later in learning [21, 22]. These findings suggest information translocation as a critical step in the cognitive processing associated with probabilistic learning.

Earlier studies found improvement in task performance with sleep and a positive correlation between immediate post-training performance and the amount of REM sleep participants obtained during the intervening night [20], linking REM sleep in this sleep-dependent improvement. Our earlier findings also suggest that, for probabilistic learning, sleep-dependent enhancements are REM dependent and remain sensitive to post-sleep interference after a short period of post-sleep wake [19].

In the current study, we used a wake-reactivation paradigm to compare immediate post-training performance with performance on the task four hours later. We also investigate the impact of interference with and without wake reactivation, during this period, to assess potential memory destabilization. The circadian timing of these tests was designed to match that of our earlier nap study, eliminating possible confounds due to circadian phase and time of day when comparing REM sleep effects seen earlier and wake effects investigated here.

## 2. METHODS

### 2.1 Participants

Eighty-eight healthy university affiliates (43 female) took part in the study in exchange for payment. Participants were between the ages of 18 and 32 (23.3 ± 3.2 (SD)) with no self-reported history of drug abuse (including alcohol and narcotics) or use of psychoactive drugs, sedatives, or hypnotics, and with no reported psychiatric, neurologic, or sleep disorders. Twenty participants (29.0%) self-identified as African American/Black, 16 (23.2%) as Asian, 16 (23.2%) as Caucasian/White, 8 (11.6%) as Multi-ethnic, and 4 (5.8%) as Hispanic or Latino. Five participants (7.2%) declined to report information on race. Potential participants who reported consuming > 600 mg/day of caffeine were excluded from the study. All participants were instructed to maintain their usual sleep schedule and documented their sleep times for the three nights immediately before the study (three-night mean sleep duration: Reactivation groups: 8.0 ± 1.1 hr., No Reactivation groups: 7.9 ± 1.3 hr., *p* = 0.61). The study was approved by the Committee on the Use of Human Subjects, Harvard University Area Institutional Review Board. All participants provided written informed consent.

Of the 88 participants who started the study, data from 69 were considered for analysis. Nineteen were excluded from analysis who (*i*) had a training score below 96% (N=2, indicating lapses in attention) or who were (*ii*) at chance (chance range: raw score, 40.9%-59.1%, p>0.05) in their responses at either initial test or retest (N=14). Seven participants showed strong evidence of spontaneously reversing the assignment of the response keys during at least one testing session. Such inversions showed evidence of otherwise normal task learning upon “repair” by reversing keystroke responses on the affected tests. Participants with’repaired’ results were included in the study.

**Figure 2.**
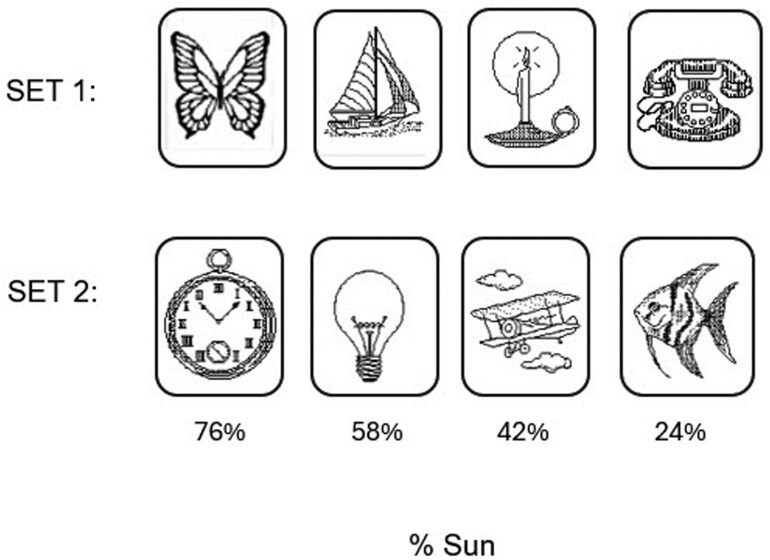
Card sets and probability of Sun for each card.

WPT stimuli came from two sets of four cards with line drawings of objects in distinct categories (animal, vehicle, light, and small device). Each card had a fixed probability of occurring with Sun or Rain, with objects from the two sets in the same category having different probabilities (Figure 2).

### 2.2 The Weather Prediction Task

#### Initial task training:

Task training consisted of 200 trials, on each of which participants were shown a card combination consisting of 1, 2 or 3 of the 4 cards from Stimulus Set 1 (Fig. 2), along with a simple image of the sun or a rain cloud indicating the weather outcome of that trial. The total of 14 possible card combinations were balanced across each of four 50 trial blocks. Prior to the start of training, participants were instructed to “attend to the cards displayed and to use the keys labeled ‘Sun’ or ‘Rain’ to enter a response matching the image of the weather condition shown immediately above the cards.” Each trial screen asked, “Rain or Sun?” If no key was pressed within 2s of stimulus presentation, the reminder, “Answer now,” appeared. After 5s the program advanced to the next trial regardless of whether an answer had been entered. Faster responses did not shorten this 5s period. The weather image presented on each training trial was determined probabilistically, based on the probabilities assigned to each of the displayed cards [15], a fact not explicitly revealed to the participants.

#### Task testing:

A 100-trial test immediately followed training, with stimuli again presented for 5s each, but without any weather symbol. Instead, participants were instructed to respond to each card combination with their ‘best guess of the weather condition predicted by the cards shown. At each trial, participants saw the prompt, “Rain or Sun?” and responded by pressing key corresponding to the chosen outcome. A response was scored as correct if the participant selected the weather choice that has the highest probability of occurring with the displayed card combination. Overall performance was scored as the percent of “correct” responses given during a test session. In all analyses, raw performance, the fraction of correct responses, is adjusted for chance occurrences of correct responses (adjusted score = [raw score - 0.5]/0.5) [23–25].

#### Reactivation task:

The reactivation task consisted of a randomized sequence of presentations of each of the 14 possible card combinations consisting of 1 to 3 of the 4 cards from Stimulus Set 1, each combination shown only once, without any weather symbol. Participants were instructed to respond to each presentation by simply indicating how many cards were showing by pressing the appropriate number key.

#### Interference training and testing:

Interference training was identical to the initial task training except that it used Stimulus Set 2 (Fig. 2). The procedure for delivering the 200 training trials and 100 trial test was otherwise identical to that of the initial training and test.

### 2.3 Experimental Protocol

Immediately before initial training, participants completed the Epworth Sleepiness Scale (ESS) [26]. Before initial testing and following retest, participants completed the Stanford Sleepiness Scale (SSS) [27], a standard measure of state subjective sleepiness with a response range of 1 (“alert, wide awake”) to 7 (“sleep onset soon”), and provided additional subjective answers at both time points to written questions on their ability to concentrate and how refreshed they felt.

**Figure 3.**
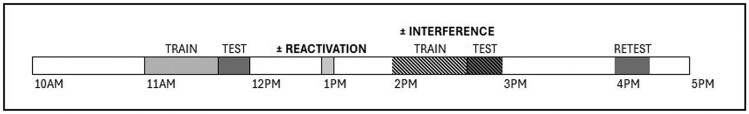
Experimental protocol. Participants underwent training and immediate testing on the WPT at 11AM and were retested at 4PM. Half of the participants had reactivation exposure at 1PM, while half of the + Reactivation participants and half of the non-Reactivation participants underwent interference training at 2PM.

Participants came to the lab and trained on the WPT at 11AM and were tested on the 100-trial task immediately following a 200-trial training (Fig. 3). The training session is therefore twice as long as the test session, as indicated on the timeline. Participants were then assigned to one of four experimental groups (-Interference, -Reactivation (-I,-R); -Interference, + Reactivation (-I,+R); + Interference, -Reactivation (+ I,-R); and + Interference, + Reactivation (+ I,+R)).

To minimize differences across the groups in mean scores on the initial test, this assignment was done algorithmically. Once a participant completed their initial test, their initial test score was compared to the current mean initial scores for the four groups, and they were added to a group so as to minimize the differences in group means while not distorting the number in each group. Code for this algorithm, which can be integrated via a TCP/IP connection into computerized tasks to balance groups for initial performance, is available at https://github.com/chhotii-alex/prospective-randomizer.

At 1PM, participants assigned to Reactivation groups performed the reactivation task. Non-Reactivation groups watched emotionally neutral videos during this interval. At 2PM, half of the participants underwent interference training, while, again, the other half watched neutral videos. All participants were then retested on the original card set at 4PM. Performance was computed as the percentage of optimal weather predictions made at training and retest. After removal of initially excluded participants (see Methods), group sizes were (-I,-R): 17; (-I,+R): 17; (+ I,-R): 18; (+ I,+R): 17.

### 2.4 Statistical Analyses

Comparative group analyses of experimental variables were carried out using ANOVA, as well as unpaired Students t-tests (two-tailed). All analyses were performed in SPSS.

## 3. RESULTS

### 3.1 Subjective alertness

SSS questionnaires to measure alertness were given twice to all groups: once before training, and again before retest These tests showed no significant group differences in ability to concentrate, how refreshed participants felt, or SSS scores (all *p*-values > 0.05), although a trend toward differences were observed in SSS scores at PM retest between the Reactivation and No Reactivation groups (for this comparison, see Table 1).

**Table 1.** Subjective alertness responses. Reactivation and No Reactivation values represent the total responses for groups (+ R, ± I) and (-R, ± I), respectively. Values = mean ± sem. SSS = Stanford Sleepiness Scale.

	Reactivation	No Reactivation	*p*
SSS (Training)	2.0 ± 0.16	2.1 ± 0.13	0.75
SSS (Retest)	2.7 ± 0.21	2.2 ± 0.18	0.073
Concentrate (Training)	71.20 ± 3.40	77.5 ± 3.30	0.19
Concentrate (Retest)	69.63 ± 2.88	76.33 ± 3.02	0.11
Refreshed (Training)	67.73 ± 3.94	72.88 ± 3.77	0.36
Refreshed (Retest)	66.4 ± 2.96	71.76 ± 2.87	0.2

### 3.2. WPT performance.

Performance on the initial, post-training test was virtually identical for the four groups (group averages = 79.5–82.3% correct; one-way ANOVA, p = 0.84; Table 2), with all groups achieving a relatively high level of performance on the morning test, immediately after training.

**Table 2.** Performance by group. Units are in average adjusted absolute score or percent improvement (of adjusted score); Values = mean ± sem.

	- Reactivation - Interference	- Reactivation + Interference	+ Reactivation - Interference	+ Reactivation + Interference
N	17	17	17	18
Initial test (Test 1)	81.8 ± 2.34	81.9 ± 2.14	82.3 ± 1.81	79.5 ± 2.89
Retest (Test 2)	83.2 ± 2.31	81.3 ± 2.71	81.3 ± 2.06	73.5 ± 4.55
Absolute improvement (Test 2 - Test1)	1.39 ± 1.21	-0.64 ± 2.73	-0.94 ± 1.68	-6.02 ± 2.84
Percent improvement (Test 2—Test 1)	1.99 ± 1.62	-0.11 ± 3.7	-0.94 ± 2	- 8.49 ± 3.49
* SD (absolute)*	5.01	11.3	6.93	12.1
* (percent)*	6.77	15.3	8.25	14.8
* p (abs.)*	0. 27	0.82	0.58	0.049*
* (%)*	0.24	0.98	0.64	0.026*
* t (abs.)*	1.1	-0.64	-0.56	-2.1
* (%)*	1.2	-0.11	-0.47	-2.4
* d (abs.)*	0.55	0.32	0.28	1.0
* (%)*	0.60	0.06	0.24	1.2

At PM retest, later in the afternoon, we observed no significant change in performance for three of the four groups: (-I,-R) p = 0.27 for absolute change, p = 0.24 for percent change; (+ I,-R) p = 0.82 for absolute change, p = 0.98 for percent change; (-I,+R) p = 0.58 for absolute change, p = 0.64 for percent change. The effect sizes associated with each of these three groups as measured by Cohen’s *d* suggested a small to medium effect: (-I,-R) d = 0.55 for absolute change, d = 0.60 for percent change; (+ I,-R) d = 0.32 for absolute change, d = 0.06 for percent change; (-I,+R) d = 0.28 for absolute change, d = 0.24 for percent change (Table 2). Retest results for those participants whose spontaneously reversed responses were “repaired” were included in the group averages (range: 64.5-89.3% correct).

In contrast to these results, participants in the (+ R,+I) group showed significantly reduced scores at retest (-6.0 ± 2.9%, p = 0.049 for absolute change; -8.5 ± 3.5%, p = 0.026 for percent change). The effect size as measured by Cohen’s *d* suggested a large effect (d = 1.0 for absolute change; d = 1.2 for percent change). Further, the decrease in performance observed in the (+ R, + I) group was significantly different from the other three groups (one-way ANOVA, absolute change: p < 0.037; percent change: p < 0.022; post hoc comparisons, post hoc p’s < 0.05 for both percent and absolute change in the (+ R,+I) group against the other three groups in multiple comparisons [LSD corrected]).

## 4. DISCUSSION

Memories can persist for years, even decades, offering the illusion that they are essentially unchanging. But this perception is misleading: both in the hours following learning and on subsequent reactivation, whether across sleep or wake, memories may become relatively labile and change dramatically [28]. During this state of lability, memories can be strengthened, modified, or even weakened, until the window for modification again closes and the memory can be considered consolidated (stabilized) again, or indeed, *re*consolidated as the cycle repeats over time [6, 28–30].

In some cases, stabilization has been observed within 4hr after encoding, independent of sleep [29, 30], while in other cases, stabilization requires post-encoding sleep [31, 32]. In contrast, improvements in task performance are generally seen only following sleep [12, 13, 33].

Those memories that see improvement with sleep appear to be affected differentially across sleep stages, depending on memory type, the mechanisms remaining unknown. Broadly speaking, slow wave sleep (SWS) appears to facilitate consolidation of declarative memory [34] and REM sleep of nondeclarative memory [5, 35]. The implicit (non-declarative)-memory-based WPT shows a correlation between evening performance on the WPT and %REM sleep on the following night [23] and between post-sleep performance improvement and REM on the intervening night [19], where the mechanism for post-sleep enhancement in REM is thought to reside with reactivation of task memory and its subsequent reconsolidation [15, 16]. Yet, reactivation can also occur during wake, where cues generating the reactivation are derived from events experienced during initial learning [36].

**Fig. 4.**
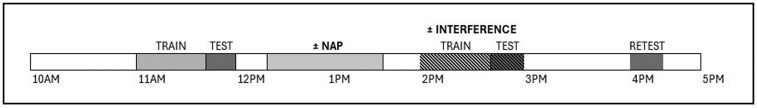
**Experimental protocol of our earlier nap study** [19]. Subjects underwent training and immediate testing on the WPT at 11 AM and were retested at 4 PM. Half of the participants had a 90-min. nap opportunity, while half of the Wake participants and half of the Nap participants underwent interference training (without subsequent testing) at 2PM.

**Figure 5.**
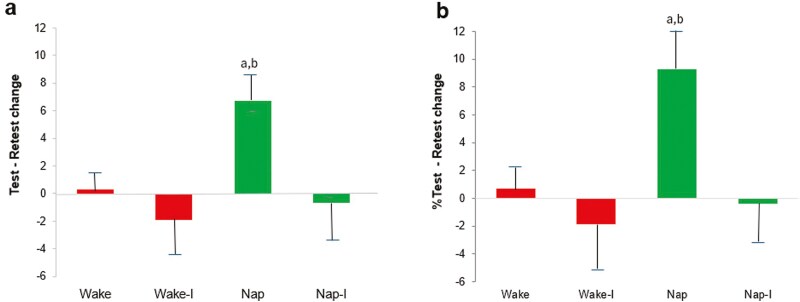
**Results of our earlier nap study** [19]. Change in adjusted score from initial testing to retest. Left: Absolute change—a The Nap group performed significantly better at Retest compared to Initial Test (p = 0.005 for absolute and % change); and b performed significantly better than all other groups (all p‘s< 0.05); Right: Percent change.

We asked in a previous study (Figs 4, 5) whether a 90-min post-encoding nap would produce REM-correlated improvements in task performance, and whether such sleep would additionally stabilize these memories, increasing their resistance to retroactive interference [19]. Our previous findings provide context to this study in that they showed that a 90-min nap is sufficient to produce an absolute improvement in WPT performance, and that the magnitude of this improvement depends on the amount of REM sleep obtained, suggesting that processes active during REM sleep enhance WPT performance. In contrast, a similar period of time spent awake produced no improvement in performance, suggesting that these processes are, in fact, sleep-dependent [19] (Fig. 5).

In the same study, utilizing an interference probe, we noted a protective effect of REM against an overall loss in performance with WPT interference [19], where interference after nap reactivation eliminated only what would have been the sleep-dependent improvement (Test—Retest change for the Nap-I group). We saw no significant evidence of an overall loss in performance after interference in either the sleep or wake groups. The REM correlation with improvement seen in the Nap group also vanished in the Nap-Interference group.

This was a perplexing result, where task memory across wake remained essentially unaffected by interference while the newly strengthened associations of sleep-dependent improvement appeared to be selectively eliminated by interference.

These earlier findings suggested that the changes in memory that occurred during sleep were distinct from the initial memory formed during training, perhaps either through (i) systems consolidation promoted by sleep, in which associative links may be made between newly formed memories [37], located in a physically distinct brain region [21, 22, 28], or (ii) by synaptic/cellular differences in plasticity between newly formed memory (those changes induced by sleep) and task memory formed earlier, that would tend to prevent reconsolidation when interference training occurs close to the time of reactivation-mediated memory modification [9].

Studies supporting the theory that a shift in location of neural activation accompanies consolidation on the WPT show a relocation of neural activation from medial temporal pathways to the striatum as WPT training progresses [21], and with sleep on an auditory probabilistic task [38]. Current thinking on memory consolidation interprets this shift as increasing potential associations [1, 39] underlying the formation of schemata [6, 7, 40], in this case representing the rules underlying the WPT, although a definitive mechanism for consolidation or enhancement remains elusive.

In the present study, we sought to explore the contrasting result between the effects of the presumed reactivation during REM and an induced wake reactivation, with the understanding that we are limited in this comparative approach: First, to the *presumption* that memory reactivation takes place during REM. Also, as no definitive evidence has yet been presented that reactivation is the critical mechanism of memory enhancement, we used differences in post-wake and post-sleep performance measures to infer that distinct processes may be at work.

We employed a wake reactivation / interference study design (Fig. 3) based on that of our earlier nap study (Fig. 4), with wake reactivation at 1PM, aligning in time with the midpoint of the nap opportunity in the earlier study, to minimize any circadian effects and to keep the encoding-to-reactivation time interval as similar as possible across the experiments.

**Figure 6.**
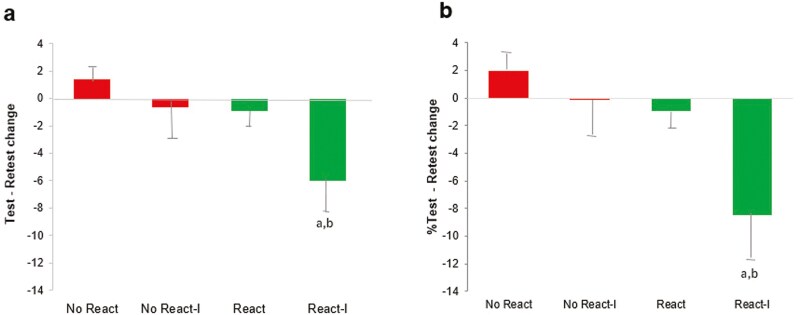
**Results of the current wake study.** Change in adjusted score from initial testing to retest. (a) Absolute change. The + Reactivation, + Interference (+ R,+I) group performed significantly worse at Retest compared to Initial Test (absolute change, p = 0.049; percent change, p = 0.026); (b) Percent test-retest change. The (+ R,+I) group performed significantly worse than all other groups (absolute change: p < 0.037; percent change: p < 0.022); Right: Percent change.

Our results indicate that wake reactivation was insufficient to produce the enhancement seen with REM, leaving unexplained differences between wake (Fig. 6) and sleep (Fig. 5) in the reactivation-consolidation process.

A nap with presumed REM reactivation improved performance (Fig. 5a), whereas wake reactivation did not (Fig. 6a). Introducing interference after wake reactivation caused a drop in performance (Fig. 6a), while interference after napping did not (Fig. 5a). We interpret the drop in performance with interference after wake reactivation as indicating that wake reactivation was indeed successful in relabilizing WPT memory, while at the same time making it susceptible to wake interference. (After a similar period of wake without induced reactivation, we saw no effect of interference on performance, which was unchanged from initial testing to retest.) This is in contrast to the effect of interference following sleep, where our earlier results suggest that REM reactivation works differently, providing protection for the original memory trace against subsequent interference, but leaving the REM-sleep-dependent enhancement of that memory susceptible (Fig. 5). These differential interference effects may offer evidence of as yet undescribed memory processing differences during or following reactivation across sleep and wake. Related studies show reciprocal interference effects between declarative (explicit) and non-declarative (implicit) memory systems [41], with generally separate patterns of wake-sleep consolidation seen in each system [9, 42, 43], and a differential response to interference seen across wake and sleep and between implicit memory, closely aligned with REM sleep, and declarative memory, associated with improvement over SWS [41, 44]. Improvement seen after REM sleep in the WPT aligns with this general pattern and is in consonance with the broadly positive association with the REM sleep stage in problem-solving, although SWS has also been associated with certain problem-solving tasks; the particular related sleep stage is thought to be dependent on memory type or the degree of explicit awareness of intention [1, 45].

Additional studies suggest that presumed reactivation during REM can contribute to the selectivity of *what* memory is consolidated [36]. Targeted memory reactivation (TMR) for explicit memories applied during REM suggest that REM sleep can decrease arousal on subsequent recall of emotional memory [46, 47] or reduce memory for low-value associations [36], suggesting a role for REM in selective pruning of memories, perhaps sharpening memories of higher value. However, no improvement of the sleep effect was seen in memory reactivated using TMR for all problem-solving tasks [48]. Though the processes that make up memory reactivation-consolidation across wake and sleep remain opaque, we found distinctly different responses to reactivation depending on conscious state: in wake, reactivation alone produces no appreciable memory improvement and, at least in the short-term, leaves post-encoding memory vulnerable to interference. In contrast, reactivation in REM sleep supports memory enhancement that is uniquely susceptible to interference.

The locus of activity during reactivation appears to be different in sleep and wake, at least as described in studies centered on explicit memory [8, 49]. Diekelmann [8] showed through fMRI evidence in an explicit memory task that reactivation during SWS was stabilizing and accompanied by activation in posterior cortical regions, while reactivation during wake was destabilizing and activated prefrontal regions. Cordi [49] further suggested that destabilizing reactivation during sleep supports memory relocation to cortical regions, increasing associative connectivity. As expected for explicit memory tasks, neither study found a correlation with REM. Whether the same pattern of REM sleep improvement and differential lability we observed across sleep and wake exists more broadly with implicit memory learning requires further investigation. And whether our observations are due to differences in the timing of neural reorganization between post-learning memory and sleep-enhanced memory [29, 30], or differences in the locus of reactivation activity remains unknown [8]. Exploration of the phenomenon of spontaneous response reversal in the WPT may yield additional information on probabilistic learning. While those participants who reversed their responses were clearly responding well to task learning on an absolute level, the possibility exists that probabilistic reversal learning may have played a part in their spontaneous key-press reversal. Interestingly, neural mechanisms for both probabilistic learning and probabilistic reversal learning appear to utilize similar pathways [4, 50].

On a different note, studies comparing pre- and post-intervention performance between groups often face a challenge in interpretation when the baseline group performance differs. To what extent are any post-intervention differences a result of the intervention or are accidental differences due to randomized assignment to groups? This issue particularly afflicts small-n studies—larger groups would tend to reduce any sampling error but are often impractical. In the present study, we utilized a novel method of prospective randomization to ameliorate this problem and keep the initial scores closely aligned. This had the desired effect of balancing lower- and higher-performing subjects across groups, affording us greater confidence that the differences we observed at retest were due to our interventions and not an epiphenomenon of the group assignment process.

### 4.1 Conclusion

For probabilistic learning, wake reactivation is insufficient for producing the enhancement seen with REM sleep. Furthermore, memory reactivated in wake is sensitive to post-reactivation interference during continued wake. This is markedly different from the interference effects seen after REM sleep, which appear to exhibit selective sensitivity in its effect on enhanced memory.

## Data Availability

The data that support the findings of this study are openly available in the Harvard Dataverse at https://doi.org/10.7910/DVN/IXFIEU

## Notes

*This paper is part of the Festschrift in honor of Dr. Robert Stickgold Collection*.
